# Sustainable E-Health: Energy-Efficient Tiny AI for Epileptic Seizure Detection via EEG

**DOI:** 10.1177/11795972241283101

**Published:** 2025-08-10

**Authors:** Moez Hizem, Mohamed Ould-Elhassen Aoueileyine, Samir Brahim Belhaouari, Abdelfatteh EL Omri, Ridha Bouallegue

**Affiliations:** 1 Innov’COM Laboratory, Higher School of Communication of Tunis, University of Carthage, Tunis, Tunisia; 2 Division of Information and Computing Technology, College of Science and Engineering, Hamad Bin Khalifa University, Doha, Qatar; 3 Surgical Research Section, Department of Surgery, Hamad Medical Corporation, Doha, Qatar; 4 Clinical Advancement Department, Hamad Medical Corporation, Doha, Qatar; 5 Vice President Medical and Health Sciences Office, QU-Health Sector, Qatar University, Doha, Qatar

**Keywords:** e-health, electroencephalography, embedded systems, epileptic seizure, IoT, machine learning, TinyML

## Abstract

Tiny Artificial Intelligence (Tiny AI) is transforming resource-constrained embedded systems, particularly in e-health applications, by introducing a shift in Tiny Machine Learning (TinyML) and its integration with the Internet of Things (IoT). Unlike conventional machine learning (ML), which demands substantial processing power, TinyML strategically delegates processing requirements to the cloud infrastructure, allowing lightweight models to run on embedded devices. This study aimed to (i) Develop a TinyML workflow that details the steps for model creation and deployment in resource-constrained environments and (ii) apply the workflow to e-health applications for the real-time detection of epileptic seizures using electroencephalography (EEG) data. The methodology employs a dataset of 4097 EEG recordings per patient, each 23.5 seconds long, from 500 patients, to develop a robust and resilient model. The model was deployed using TinyML on microcontrollers tailored to hardware with limited resources. TensorFlow Lite (TFLite) efficiently runs ML models on small devices, such wearables. Simulation outcomes demonstrated significant performance, particularly in predicting epileptic seizures, with the ExtraTrees Classifier achieving a notable 99.6% Area Under the Curve (AUC) on the validation set. Because of its superior performance, the ExtraTrees Classifier was selected as the preferred model. For the optimized TinyML model, the accuracy remained practically unchanged, whereas inference time was significantly reduced. Additionally, the converted model had a smaller size of 256 KB, approximately ten times smaller, making it suitable for microcontrollers with a capacity of no more than 1 MB.

These findings highlight the potential of TinyML to significantly enhance healthcare applications by enabling real-time, energy-efficient decision-making directly on local devices. This is especially valuable in scenarios with limited computing resources or during emergencies, as it reduces latency, ensures privacy, and operates without reliance on cloud infrastructure. Moreover, by reducing the size of training datasets needed, TinyML helps lower overall costs and minimizes the risk of overfitting, making it an even more cost-effective and reliable solution for healthcare innovations.

## Introduction

A few people have witnessed someone experiencing sudden seizures, characterized by distinctive movements. These occurrences can occasionally result in significant complications that affect both physical and mental well-being, a condition termed epileptic seizures by neurologists. Epilepsy is a chronic neurological disorder that affects approximately 50 million people worldwide. This results in permanent seizures, where abnormal brain activity triggers episodes of unusual behavior, occasionally followed by a fainting and impaired control over the bladder or blower functions.^1^

For a long time, neuroscientists believed that epileptic seizures would spark suddenly, just moments before observable clinical manifestations. However, recent advancements have enabled scientists to monitor the brain activity of a particular region and cell type, which enables the prediction of epileptic seizures before their visible onset. Research suggests that with reliable seizure prediction, individuals with epilepsy could proactively take appropriate preventative measures, by adjusting their activities, activating neurostimulators, or promptly taking medications to avert or mitigate the impact of a seizure. The key to achieving swift and effective epileptic crisis prediction lies in the utilization of data and monitoring devices gathered from the IoT ecosystem, coupled with the application of artificial intelligence (AI).^2^

Electroencephalography (EEG) remains a crucial and indispensable tool in the medical field for detecting the initial signs of illness in patients with epilepsy (PWE), Its proven contribution to the evaluation, diagnosis and classification of epileptic seizures is well-established.^3-7^

In the detection and prediction of epileptic seizures,^8^ various techniques have been established, primarily focusing on EEG signals. These methods incorporate diverse signal analysis approaches, including AI techniques such as ML algorithms with neural networks.^9^ Notably, these techniques are applied in implanted medical devices such as neurostimulators, utilizing intracranial EEG (iEEG) for seizure detection and delivering electrical impulses to prevent seizures.^10^ Another application involves the assessment of EEG signals in portable devices, such as bands or watches, which serve to alert patients to an imminent seizure.^11^

The Internet of Things (IoT) is recognized as a pivotal component of clinical care, offering substantial solutions for diverse clinical and healthcare applications. IoT technologies enable the continuous and real-time monitoring of patients’ health through wearable devices. These methods are also used to capture and transmit EEG signals from patients. Concurrently, ML algorithms provide potential solutions for the effective detection of epileptic seizures from EEG signals extracted from electrodes. When integrated with AI algorithms, IoT has emerged as a promising technology to address various challenges in the medical care domain. To achieve early identification of epileptic seizures, it is imperative to develop an automatic framework that utilizes current communication technologies in conjunction with IoT, ML, and cloud computing.^12^

Recent advancements in epileptic seizure detection have utilized innovative machine ML and Deep Learning (DL) techniques, significantly enhancing both the accuracy and efficiency in identifying and predicting seizure events. For early epilepsy detection, effective methods were introduced to extract significant features and classification using technologies such as the discrete wavelet transform (DWT), *t*-distributed stochastic neighbor embedding (t-SNE), K-means clustering, and K-Nearest Neighbors (K-NN). These techniques contribute to more accurate and timely detection of seizures.^13-18^ In addition, different practical ML algorithms have been employed to classify EEG patterns in healthcare applications, such as electrical brain movement,^19^ emotional states while listening to music,^20^ cardio-oncology,^21^ eye movements,^22^ deciphering speech,^23^ mental disorders,^24^ and wearable biofeedback system.^25^ Moreover, many studies have introduced a range of techniques that are contingent upon different features and classifiers. These methods comprehensively elucidate seizure predictions and offer insights and avenues for future research.^26-32^

**Figure 1.**
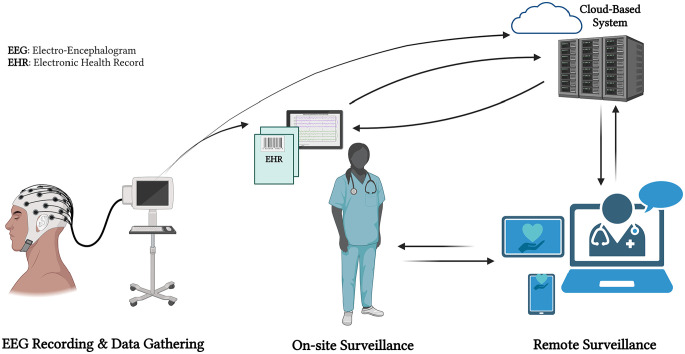
Internet of medical things (Created with Biorender.com).

Despite recent advancements in epileptic seizure prediction and detection, some problems remain unresolved. Many of the available methods require powerful and expensive infrastructure platforms. Some solutions use cloud computing, which can be slow when quick responses are needed, which makes them difficult to use in small, portable devices, and in resource-limited environments, making them non-inclusive technologies. Combining new prediction methods with IoT devices for constant monitoring and quick alerts remains difficult because we need to develop lightweight models suitable for deloployment on small devices with limited memory and processing power. Few studies have examined the use of TinyML for epileptic seizure prediction as IoT and e-health applications.^33,34^ Figure 1 provides a detailed overview of the Internet of Things (IoT) and e-health applications specifically designed for epileptic seizure prediction based on EEG signals. This illustrates how IoT devices are integrated with e-health platforms to continuously monitor and analyze EEG data in real-time, enabling the prediction of epileptic seizures and enhancing patient care.

The current study aimed to (i) develop a TinyML workflow that outlines the steps for designing and deploying models in resource-constrained environments, and (ii) apply this workflow to e-health applications for real-time epileptic seizure detection using EEG data.

## Methodology

### Definitions

#### Internet of Things (IoT)

An IoT system is designed for automatic data exchange, and it typically includes sensors, actuators, and software devices. Its applications are diverse and include agricultural machinery, such as tractors, irrigation systems, and drones, as well as medical wearables, such as wristbands that monitor blood pressure and heart rate, and glucometers. The IoT also encompasses everyday items such as connected cars and devices that can acquire, process, and exchange data related to controlled environments or disseminate information. The IoT landscape is constantly evolving, and numerous applications under development are expected to have a significant impact on various aspects of our lives. Connected health is one of the most advanced domains within the IoT landscape, and it is expected to revolutionize healthcare.^2^

#### e-health

e-health is the primary domain in which IoT applications have had the most significant influence. According to Scarpato et al,^2^ digital methods are utilized to enhance healthcare services. Over the past decade, this application has undergone substantial development due to the remarkable advancements in AI algorithms.

#### AI for IoT and e-health

AI specifically refers to the creation of intelligent computer programs. It involves performing out tasks typically associated with intelligent life forms. Unlike traditional software systems, it focuses on enhancing the intelligence of a computer system through statistical learning algorithms. It outlines the process of decision making using training algorithms.^35^

#### ML in e-health

E-health is a rapidly growing field in the application of ML. With the digitization of health services, vast amounts of data have been generated over time. To ensure accurate diagnoses and improve patient outcomes, it is crucial to utilize ML algorithms effectively while avoiding incorrect predictions and protecting patient privacy. However, this presents a significant challenge for researchers working in the field of ML applications in e-health.^2^

#### ML for epileptic seizure prediction

A major application in the E-health domain involves the early detection and prediction of various diseases, including epileptic seizures. Thanks to advances in ML algorithms and the expansion of epilepsy databases, researchers are now able to accurately predict seizures in their early stages. ML plays a crucial role in the treatment of epilepsy by analyzing electroencephalography (EEG) to predict seizures and identify the affected region of the brain, as well as medical outcomes.^36-38^

#### TinyML in E-health

Edge computing, particularly TinyML, has emerged as a promising solution for improved performance,^39^ with its ability to ensure high speed and respect privacy. TinyML merges ML with microcontrollers and IoT devices, enabling local analytics and leveraging the large quantities of data collected. However, challenges may arise at the interface, such as troubleshooting difficulties and limited memory constraints.^40-42^

### Problem formulation

This study addresses the significant challenges in characterizing brain diseases, with a particular focus on epileptic seizures. Traditional diagnostic methods often fail to effectively harness the complexity of EEG data, leading to limitations in accuracy and real-time applicability. The pressing need for sophisticated yet computationally efficient solutions is evident because conventional ML approaches typically require substantial processing power, making them unsuitable for resource-constrained devices. In addition, the latency associated with cloud-based processing hinders timely intervention in critical medical scenarios. The core challenge of this study lies in developing a novel e-health application that seamlessly integrates EEG data with TinyML, specifically designed to address the complex task of epileptic seizure detection.

The deployment of TinyML models on embedded devices enables the real-time processing and analysis of EEG data directly on the device without the need for continuous data transmission to a cloud server, which can be slow and costly. The proposed method processes the EEG data locally on the microcontroller, avoiding the delays associated with data transmission and remote processing. This approach enables faster response times than those of cloud-based solutions. Localized processing ensures that personal health data remains on the device, reducing the risk of data breaches and unauthorized access.

To overcome these limitations and bridge the gap with existing methods, we conducted a comparative study (Table 1) alongside previous research that utilized traditional ML on clouds,^33^ edge computing with ML,^33^ and wearable devices with basic analytics.^34^ The comparison focused on key aspects such as computational efficiency, real-time processing, privacy and security, cost-effectiveness, robustness and adaptability, and real-world validation.

**Table 1.** Comparison of the current study with methods used in the literature.^33-34^

Criteria	Proposed method (Tiny AI/TinyML for EEG and E-Health)	Method 1 (Traditional ML on cloud)^33^	Method 2 (Edge computing with ML)^33^	Method 3 (Wearable devices with basic analytics)^34^
Computational efficiency	High efficiency on resource-constrained devices	High resource demand, relies on cloud resources	Moderate efficiency, requires more powerful edge devices	Limited processing capabilities
Real-time processing	Yes, low latency	No, depends on network speed and cloud processing time	Yes, but dependent on edge device capabilities	Limited, mainly basic analytics
Privacy and security	High, data processed locally	Lower, data sent to cloud for processing	Moderate, data processed at the edge but may still transmit some data	High, data remains on the device
Cost-effectiveness	High, no ongoing cloud costs	Low, continuous cloud service costs	Moderate, initial cost high but lower operational costs	High, low ongoing costs
Robustness and adaptability	High, adaptable to various devices	Moderate, depends on cloud infrastructure	High, adaptable but depends on edge device specs	Low, limited to basic functions and specific hardware
Real-world validation	Yes, tested with real-world epilepsy dataset	Varies, often based on synthetic datasets	Yes, but less common than cloud methods	Limited, often not validated with comprehensive datasets

### Data acquisition

In this study, data acquisition is a critical component of the ML process. The choice to utilize the Bonn EEG database^43^ was driven by the limited availability of epilepsy-related data, coupled with the database’s extensive recognition and use in the field. This database comprises 4097 EEG recordings per patient, each spanning 23.5 seconds, with a total of 500 patients. To facilitate the analysis, we divided the 4097 data points into 23 segments per patient, resulting in a dataset with 11,500 rows and 179 columns. Each row represents patient’s EEG data, with 178 columns corresponding to EEG readings collected over 1 second, and the final column indicates the patient’s seizure status.

Feature engineering typically involves converting categorical or ordinal features into numerical variables that are suitable for ML algorithms. However, in the case of this epilepsy dataset, the 178 columns directly represent EEG readings captured at specific time points. Because these readings provide raw numerical data essential for analysis, further feature engineering is considered unnecessary.

### Algorithm and model selection

**Figure 2.**
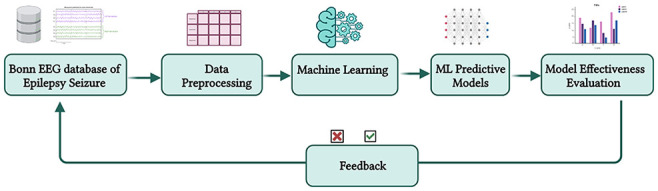
ML workflow for data collection, training, and deployment (Created with Biorender.com).

Before considering the use of ML to predict epileptic seizures, it is essential to examine whether ML represents the optimal approach for this challenge. Figure 2 depicts the ML workflow adopted for epileptic seizure prediction and characterization. This process involves using patient-derived data as input to the ML system, facilitating the analysis of this data and generating predictions in real-time.

In this study, various algorithms and models, such as K-nearest neighbors (KNN), logistic regression, Stochastic Gradient Descent (SGD), naive Bayes, decision tree classifier, random forest, gradient boosting classifier, extremely random trees, and XGBoost classifier, were examined to assess their performance and identify the most suitable one for our study.

■ KNN: This is a classification technique that assigns a sample to the class most frequently occurring among its K closest neighbors in the feature space.^44^
■ Logistic Regression (LR): This is a classification model that predicts probabilities by mapping input features to a linear equation.^45^
■ SGD: This is a versatile optimization algorithm used in linear regression, logistic regression, and clustering models.^46^
■ Naïve Bayes: This approach computes feature probabilities per class, multiplies and normalizes them to classify a sample into the class with the highest probability.^47^
■ Decision Tree Classifier: The classification algorithm iteratively divides data into subregions until all samples belong to pure classes or meet certain criteria.^48^
■ Random Forest: It is built by aggregating decision trees using bagging, ensuring that they are uncorrelated.^49^
■ Gradient Boosting Classifier: It starts with an initial prediction for each individual, represented by a leaf.^50^
■ Extremely Random Trees: This is like a Random Forest, except that it selects variables for splitting by drawing samples from the entire training set.^51^
■ XGBoost Classifier: This similar to gradient boosting but utilizes trees with varying terminal nodes and a more regularized model to prevent overfitting.^52^

### Key evaluation metrics

The model’s performance was evaluated using various metrics on the dataset. These key evaluation metrics offer a comprehensive view of the model’s effectiveness in making accurate predictions and its computational efficiency. The evaluation metrics included specificity, prevalence, confusion matrix, accuracy, precision, recall, and the F1 score. These metrics can be calculated as follows:

(1) $$Specifity=\frac{TN}{TN+FP}$$

(2) $$Prevalence=\frac{Number\;of\;positive\;instances}{Total\;number\;of\;instances}$$

(3) $$Confusion\;Matrix=\left[\begin{array}{cc} TP & FN\\ FP & TN \end{array}\right]$$

(4) $$Accuracy=\frac{TP+TN}{TP+FN+FP+TN}$$

(5) $$Precision=\frac{TP}{TP+FP}$$

(6) $$Recall=\frac{TP}{TP+FN}$$

(7) $$F1\;\;Score=\frac{2*Precision*Recall}{Precision+Recall}$$

Where:

*N: The number of target classes in the dataset.*

*True Positives (TP): Where the model correctly predicts the positive class.*

*False Positives (FP): Where the model correctly identifies the negative class.*

*True Negatives (TN): Where the model incorrectly identifies the negative class as positive.*

*False Negatives (FN): Where the model incorrectly identified the positive class as negative.*

In our work, we focused on AUC (Area Under the Receiver Operating Characteristic Curve), that represents the area under the ROC curve. A higher AUC indicates a better overall performance of the model. The data are divided into “Train,” “Validation,” and “Testing” segments to assess the selected model. The outcomes are compared using the AUC Learning Curve, the variation of maximum features on AUC for both the training and validation sets, and finally, the ROC curve.

### Tiny machine (TinyML) learning deployment

**Figure 3.**
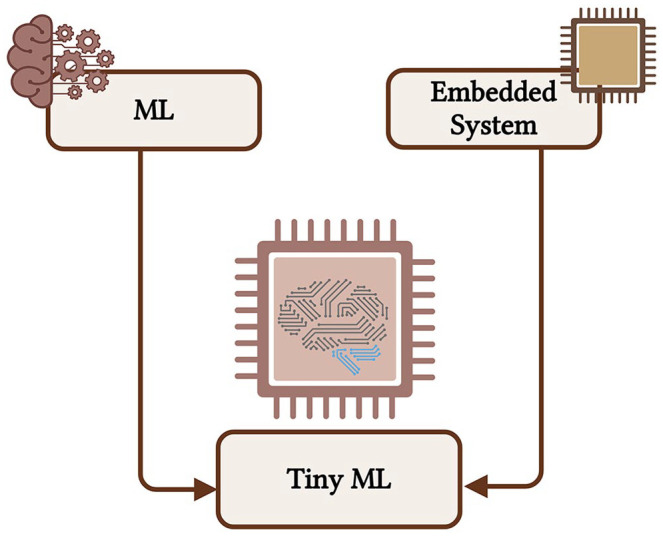
Transition from ML to TinyML (Created with Biorender.com), adapted with modifications from Aoueileyine.^59^

As the demand for intelligent applications on resource-constrained embedded systems grows, TinyML has emerged as a crucial solution for optimizing performance. As shown in Figure 3, TinyML combines ML with embedded systems to address the challenges posed by limited processing memory and power.^39^ Traditional models designed for high-performance environments can overwhelm such devices, causing latency and inefficiency.^40^ TinyML mitigates these issues by creating lightweight models tailored for constrained devices using techniques, such as quantization and model compression.^41^ This approach aligns with edge computing, enabling real-time decision-making close to the data source, which is crucial for applications in healthcare and IoT.

The key advantage of TinyML is its remarkable energy efficiency, enabling rapid inference directly on devices. This not only extends battery life but also reduces environmental impact and operational costs. Additionally, TinyML minimizes the size of the training dataset required, helping to reduce overfitting and allowing for more effective use of data. Its applications span a wide range of fields, from predictive maintenance in industrial IoT to personalized health monitoring in e-health, providing cost-effective and sustainable solutions in areas where traditional computing methods often fall short.^42^

**Figure 4.**
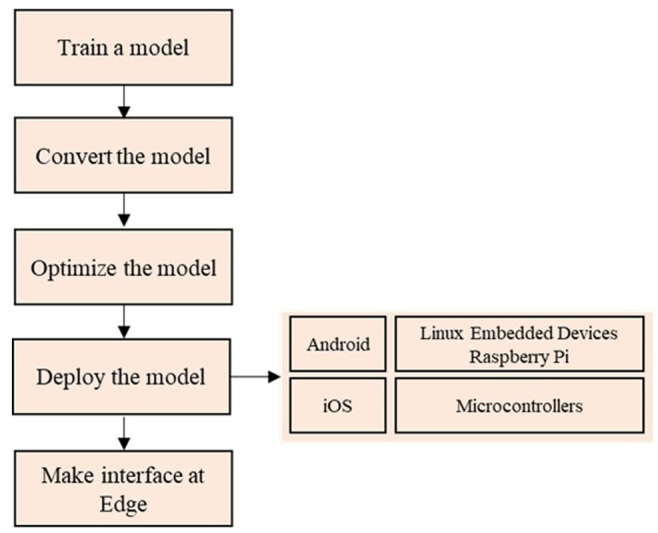
Model conversion for TinyML deployment workflow.

For the model conversion, TensorFlow Lite (TFLite) is used for running ML models efficiently on small devices, such as wearables. Figure 4 depicts the TinyML workflow using TFLite. The models are converted into TFLite format using a specified converter. Two operations reduce the model size without sacrificing performance: pruning removes connections or neurons, while quantization decreases the precision of the weight and bias values.

## Results and Discussion

In this section, we present a comprehensive evaluation of ML algorithms for epileptic seizure detection, highlighting the selection of the optimal model, performance metrics, validation techniques, and the implementation of the chosen model on constrained devices, including a detailed comparison with existing literature.

**Figure 5.**
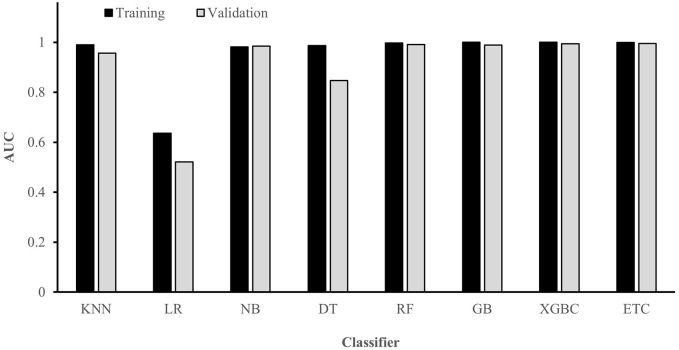
Classifier evaluation and algorithm choice.

For the choice of the best ML algorithm and based on the AUC values shown in Figure 5 for both the training and validation sets, it is evident that the ExtraTrees Classifier outperforms other models, achieving a remarkable 99.6% AUC in the validation set. Therefore, the ExtraTrees Classifier was selected as the preferred classifier.

**Figure 6.**
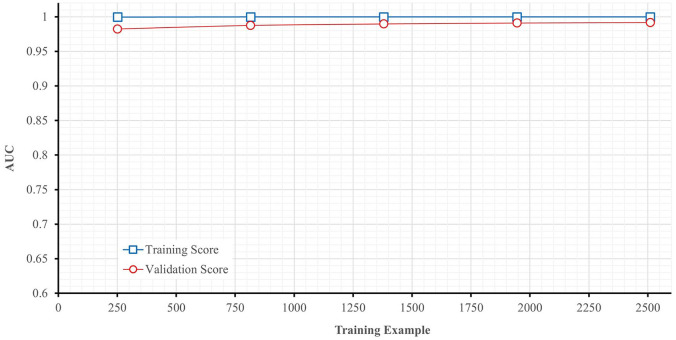
AUC learning curve for ExtraTrees classifier.

**Figure 7.**
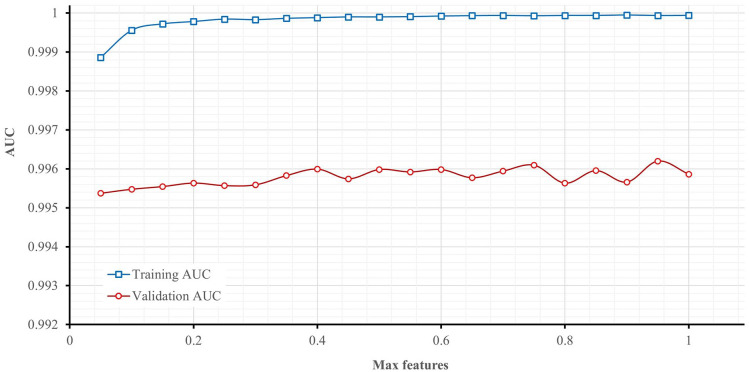
Effect of max features on AUC.

For the evaluation performance, Figure 6 gives the AUC curve variation in training and in cross validation, and Figure 7 illustrates the AUC convergence over the iteration and gives the final values in the training and validation phases.

**Figure 8.**
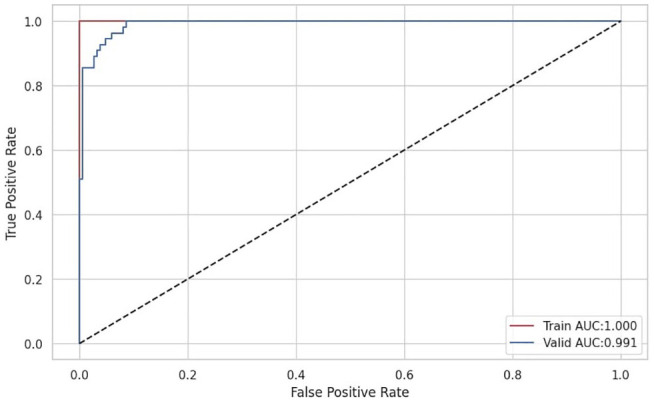
ROC curve.

For the validation, we present the ROC curve illustrated in Figure 8 which provides a comprehensive assessment of a model’s ability to discriminate between the positive and negative classes. It provides insights into the trade-offs between sensitivity and specificity at different decision thresholds. Additionally, the key performance metrics summarized in Table 2 provide an overview of the variations in the performance parameters during the training, validation, and testing phases.

**Table 2.** Key performances indicators (KPIs) for the selected model.

KPI	Training	Validation	Testing
AUC	1.00	0.997	0.997
Accuracy	1.00	0.965	0.959
Recall	1.00	0.977	0.974
Precision	1.00	0.866	0.842
Specificity	1.00	0.962	0.955
Prevalence	0.50	0.199	0.197

For the constrained device choice and evaluation, the performance of tiny microcontrollers and clouds is compared in Table 3.^53-58^ Embedded systems and connected devices provide several significant advantages, particularly in the field of e-health, leveraging ML models. TinyML enables the execution of complex ML models on microcontrollers, offering capabilities distinct from those of giant processors.

**Table 3.** Comparison of performances criteria for target devices.^53-58^

Category	Chipset	Memory size	Storage type	Energy usage	Price (USD)
Compact device (e.g., Arduino Nano 33 BLE)^53^	ARM Cortex-M4	256 kB SRAM	1MB eFlash	0.05 W	$3
Cloud platform (e.g., NVIDIA)^54^	NVIDIA Volta GPU	16 GB	SSD/Hard Disk (TB-PB)	250 W	$9,000
Microcontroller (e.g., ESP32)^55^	Xtensa LX6 Dual-Core	520 kB SRAM	4MB Flash	0.2 W	$10
Development board (e.g., Raspberry Pi 4)^56^	ARM Cortex-A72 Quad-Core	2 GB-8 GB LPDDR4	microSD (up to 256 GB)	5 W	$35-$75
IoT Device (e.g., Raspberry Pi Zero W)^57^	ARM1176JZF-S	512 MB LPDDR2	microSD	1.5 W	$10
Microcontroller (e.g., STM32F4)^58^	ARM Cortex-M4	192 kB SRAM	1 MB Flash	0.15 W	$15

**Figure 9.**
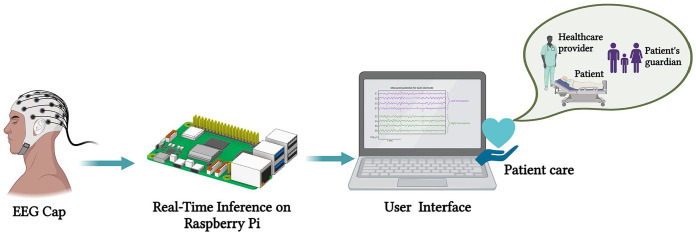
Framework for deploying TinyML solutions (Created with Biorender.com), adapted with modifications from Aoueileyine.^59^

**Figure 10.**
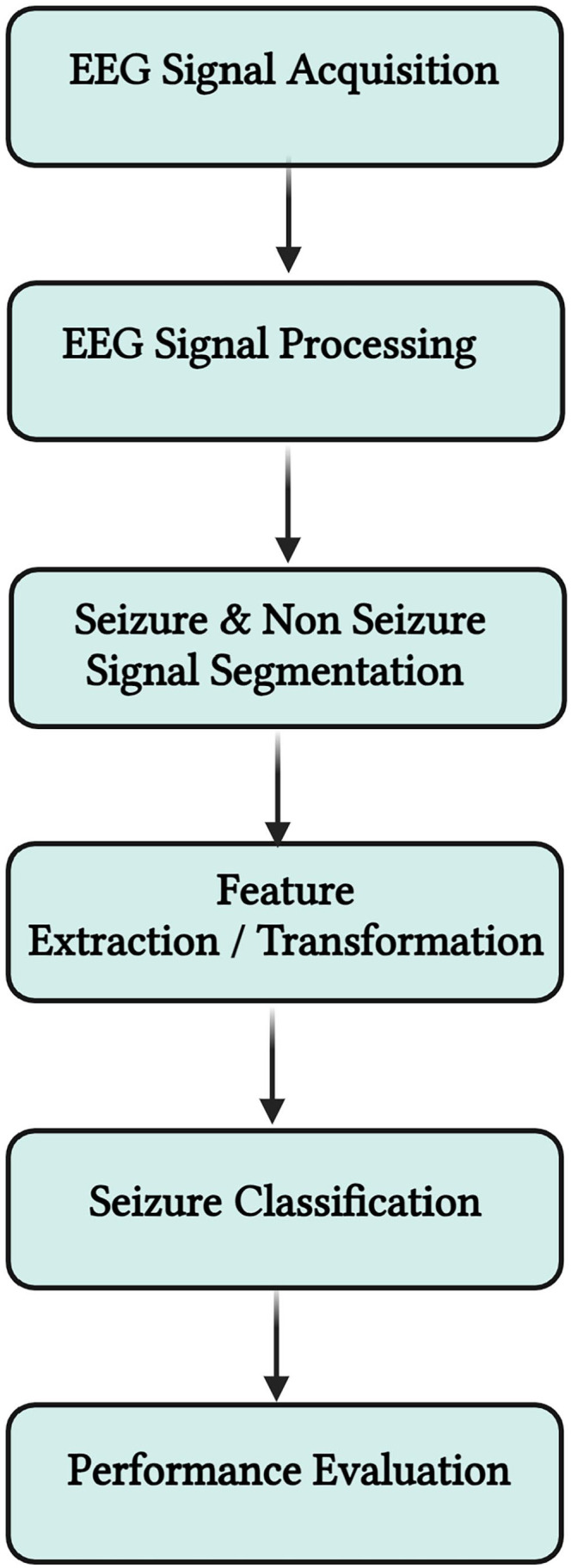
Real-time inferences on constrained performance devices (Raspberry Pi) (Created with Biorender.com).

To implement our model on the Raspberry Pi 4, it needs be converted to a TFLite format for efficient execution on the device’s firmware. Our plan is to integrate it into a small computer setup equipped with sensors that capture EEG signals, preprocess these signals, and use the trained model for predicting epileptic seizures. For evaluation, we plan to implement the model on the ARM-based Raspberry Pi 4 nano-computer. The deployment workflow is illustrated in Figure 9. The first step involves collecting brain activity data using electrodes placed on the scalp. The EEG device captures the EEG signal, which was subsequently examined and classified into “seizure” and “non-seizure” conditions. Finally, in Figure 10, features were extracted from the data to eliminate any irrelevant information, aiding the classifier in effectively discriminating between features.

TinyML converts the constraints of embedded hardware, including performance, power consumption, and storage into strengths, despite the more powerful model versions residing in the cloud. Unlike traditional ML applications that use multiple cloud TPUs or GPUs for training, TinyML facilitates efficient execution on embedded devices. In this context, the learning process does not occur on the device itself; instead, it executes previously acquired knowledge, enhancing efficiency in resource-constrained environments.

The outcomes of running two iterations of the optimized model are presented: initial and converted versions. The conversion involved pruning and quantization steps, reducing the neuron count and transforming the data type to fit (int8) instead of (float32), while the accuracy remained almost unchanged. Moreover, the converted version had a smaller model size of 256 KB, a reduction of approximately 10 times, making it suitable for microcontrollers with a capacity of not more than 1 MB. In addition, the inference time was significantly reduced to 0.002185 seconds, as reported in our previous study.^59^ Table 4 compares the results of the current study with the existing literature in terms of scope, methodology, results, and contribution.^60-63^

**Table 4.** Results comparison.^60-63^

References	Scope	Methodology	Results	Contribution	Limitations
Sachadev and Bhatnagar^60^	Importance of medical imaging in diagnosing brain disorders, with a particular focus on the role of EEG signals.	The methodology includes investigating AI-based methods for feature extraction and classification using EEG data.	The potential and effectiveness of incorporating AI in analyzing EEG signals for disease mapping and predictions.	The potential and efficiency provide a comprehensive overview of the technologies and AI-based methods used in mapping brain diseases.	The complexity of analyzing EEG signals and the difficulties associated with incorporating AI models into practical medical settings.
Zubov et al^61^	Proposing a system for controlling smart home appliances through brain-to-thing communication, utilizing EEG technology, edge IoT devices, and the MQTT protocol.	Utilizing the non-invasive Sichiray TGAM brainwave EEG sensor kit to capture signals, which are then transmitted to an embedded module via Bluetooth.	The findings validate the practicality and user approval of the suggested smart home management system for individuals with mobility impairments.	Providing an innovative approach to enable individuals with mobility impairments	Requirement for additional testing involving a broader and more varied group of users.
Shin et al^62^	A wearable EEG device facilitating real-time interaction between human brain states and AI, to improve decision-making in autonomous systems.	A wireless, earbud-style EEG measurement device equipped with tattoo-like electrodes for continuous, high-quality EEG signal capture.	The efficiency of the Brain–AI Closed-Loop System demonstrated through its practical applications in AI-driven machines.	Integrating error-related potential signals improves the system’s adaptability and enhances AI decision-making capabilities.	A requirement for additional validation across varied and complex scenarios.
Shoka et al^63^	An innovative framework that integrates encryption techniques with Convolutional Neural Networks (CNNs) to protect sensitive medical EEG data used in the detection of epileptic seizures for telemedicine applications.	A framework that transforms EEG time series into 2D spectrogram images, which are then encrypted using the Chaotic Baker Map, and Arnold Transform algorithms.	The experimental findings, verified using the public CHB-MIT dataset, showcase the effectiveness of the proposed method.	The necessity for additional validation across varied datasets and the evaluation of computational efficiency for practical implementation in telemedicine applications.	The necessity for additional validation across varied datasets and the evaluation of computational efficiency for practical implementation in telemedicine applications.
Current study	TinyML and IoT technologies are optimized for embedded systems with constrained resources, with a special focus on an e-health application aimed at characterizing brain diseases.	A TinyML workflow, detailing the steps required to develop a model that is appropriate for environments with limited resources.	The effectiveness of the TinyML approach in settings with limited resources, especially for e-health applications focused on the characterization of brain diseases.	The real-world application of a TinyML workflow for characterizing brain diseases within e-health applications.	Validation across various datasets and assessment of the scalability of the TinyML approach for different brain diseases.

Embedded hardware has limited performance, power consumption, and storage, but TinyML transforms these limitations into strengths, despite the fact that the original cloud-based model is more powerful. In typical ML applications, cloud TPUs or GPUs training algorithms to learn patterns from the data. However, in TinyML, we apply pre-learned patterns to the embedded device rather than learning new ones.^40-42^

Integrating Tiny AI and TinyML into e-health applications for brain disease characterization, particularly in epileptic seizure prediction, offers a transformative approach with several benefits and challenges.^39^ The proposed method enhances computational efficiency, enabling real-time processing on resource-constrained devices such as microcontrollers. Localizing the data processing ensures low latency, which is crucial aspect for timely medical interventions. This strategy additionally enhances data privacy and security by minimizing the requirement for internet data transmission. The method’s high efficiency makes it well-suited for portable and wearable health monitoring systems. It enables quick alarms and interventions, which are crucial for controlling illnesses such as epilepsy. Timely reactions can have a major impact on patient outcomes.

The proposed method enhances computational efficiency, enabling real-time processing on resource-constrained devices such as microcontrollers. This localized data processing ensures low latency, which is crucial for timely medical interventions, and improves data privacy and security by minimizing internet data transmission. It is ideal for portable and wearable health monitoring systems, offering immediate alerts and interventions essential for managing conditions such as epilepsy, where timely responses are critical.

The method’s capacity to handle data on-site offers a convincing resolution. It greatly diminishes the likelihood of breaches and unauthorized entry, effectively dealing with the crucial matter of patient confidentiality in the healthcare sector. This technique is also cost-effective, as it decreases the dependence on cloud services and hence decreases the continuing operational expenses. This enhances the long-term sustainability of the technology, making it more economically viable for healthcare institutions to allocate resources in a more efficient manner. Moreover, the versatility of the TinyML workflow on diverse hardware platforms guarantees adaptability and scalability across a range of e-health devices, thereby broadening its potential uses and influence.

However, there are challenges that need to be addressed. The limited processing power of microcontrollers may restrict the complexity of deployable models, potentially affecting the accuracy and performance in more demanding applications. Model maintenance can be challenging, because updating and maintaining models in a distributed environment with numerous devices requires robust mechanisms to ensure consistency and reliability. Finally, data constraints from localized processing may limit the amount of data available for training and inference, potentially impacting the model’s ability to generalize from broader datasets and affecting its effectiveness in various scenarios.

The integration of TinyML with IoT in the presented e-health application opens new avenues for further exploration. Investigating the scalability and adaptability of the TinyML approach to brain diseases beyond epileptic seizures is crucial.^20^ Assessing its effectiveness across diverse datasets and neurological conditions broaden TinyML’s applicability in real-world healthcare scenarios. In addition, it is essential to explore the security and privacy implications of transmitting health data to the cloud is essential. Addressing data protection and secure communication concerns is vital for the widespread adoption of such e-health applications.^20^ Further research should focus on refining encryption methods and ensuring compliance with healthcare-data privacy standards.

The current study presents an opportunity to investigate the synergies between TinyML and other emerging healthcare technologies, including edge computing and federated learning. By examining the ways in which these technologies can collaborate, more efficient and secure eHealth applications can be developed. Furthermore, it is important to consider the energy efficiency and environmental impact of deploying TinyML models to resource-constrained devices to ensure sustainable healthcare solutions. Additionally, it is essential to pay attention to the usability and acceptance of the developed e-health application among both healthcare practitioners and patients.

Studies conducted by scholars^64-67^ have emphasized the importance of real-time processing and the potential of AI in healthcare applications. Their work highlighted the necessity for efficient, secure, and scalable solutions, which aligns with the goals of this study. Future research can build on these findings by incorporating advanced AI techniques and exploring collaborative frameworks that integrate TinyML within broader healthcare ecosystems.

Our research on TinyML for detecting epileptic seizures shows great potential, however, it is important to acknowledge certain limitations. First, although our study examined a subset of publicly available EEG recordings, validation with larger datasets and more inclusive criteria may be necessary. Additionally, although we tested our model on Raspberry Pi devices in controlled environments, conducting randomized clinical studies to evaluate its performance, as well as addressing privacy and ethical considerations, will be crucial. We also recognize the need to assess the long-term impact of continuous monitoring on the battery life and device performance, which requires further investigation. While we are optimistic about the potential of our findings, we understand that additional work is needed to ensure their practical benefit for individuals living with epilepsy and to bridge the gap between laboratory research and real-world applications. These challenges will be the primary focus of our future research.

## Conclusions

This study demonstrates how TinyML can be used to detect epileptic seizures in real-time using EEG data, focusing on creating a model that performs efficiently on small, resource-constrained devices. The ExtraTrees Classifier emerged as the best-performing model, achieving a 99.6% AUC score on the validation set. Even after optimization for deployment on tiny devices, the model maintained its accuracy while becoming significantly smaller and faster.

One of the key advantages of this work is its potential to enable continuous monitoring of individuals with epilepsy using affordable, wearable devices. These devices can quickly alert patients or caregivers to impending seizures, providing greater freedom and security in daily life. By processing data directly on the device. The system also enhances patient privacy by directly processing data on the device.

Our TinyML solution integrates well with other medical devices and systems, potentially leading to improved overall care for patients with epilepsy. This could reduce hospital visits and assist doctors in developing more effective treatment plans. The success of this project indicates that similar approaches may be beneficial for other neurological health issues.

However, further research is necessary to validate this system in real-world environments and in larger patient populations. Future studies should also assess the effectiveness of the model over extended periods and across different types of epilepsy. As we continue to refine this technology, we aim to make a meaningful impact on individuals’ lives and contribute to advancements in healthcare.
